# Follow-up of Contacts of Middle East Respiratory Syndrome Coronavirus–Infected Returning Travelers, the Netherlands, 2014

**DOI:** 10.3201/eid2109.150560

**Published:** 2015-09

**Authors:** Madelief Mollers, Marcel Jonges, Suzan D. Pas, Annemiek A. van der Eijk, Kees Dirksen, Casper Jansen, Luc B.S. Gelinck, Eliane M.S. Leyten, Ingrid Thurkow, Paul H.P. Groeneveld, Arianne B. van Gageldonk-Lafeber, Marion P. Koopmans, Aura Timen, for the MERS-CoV Outbreak Investigation Team of the Netherlands

**Affiliations:** European Center for Disease Prevention and Control, Stockholm, Sweden (M. Mollers);; National Institute for Public Health and the Environment, Bilthoven, the Netherlands (M. Mollers, M. Jonges, A.B. van Gageldonk-Lafeber, M.P. Koopmans, A. Timen);; Erasmus MC, Rotterdam, the Netherlands (M. Jonges, S.D. Pas, A.A. van der Eijk, M.P. Koopmans);; Public Health Service Haaglanden, The Hague, the Netherlands (K. Dirksen);; Medical Centre Haaglanden, The Hague (C. Jansen, L.B.S. Gelinck, E.M.S. Leyten);; Public Health Service Ijsselland, Zwolle, the Netherlands (I. Thurkow);; Isala Klinieken Zwolle, Zwolle (P.H.P. Groeneveld)

**Keywords:** MERS-CoV, Middle East respiratory syndrome coronavirus, monitoring, contact investigation, psychological effect, IES-R, knowledge, perception, serology, PCR, viruses

## Abstract

Notification of 2 imported cases of infection with Middle East respiratory syndrome coronavirus in the Netherlands triggered comprehensive monitoring of contacts. Observed low rates of virus transmission and the psychological effect of contact monitoring indicate that thoughtful assessment of close contacts is prudent and must be guided by clinical and epidemiologic risk factors.

During April 2012–May 2015, the World Health Organization received 1,110 notifications of confirmed cases of infection with Middle East respiratory syndrome coronavirus (MERS-CoV), including at least 422 deaths (*1**,**2*)*,* mostly from countries in the Arabian Peninsula. Travel-related cases have been reported in Europe, Asia, and the United States, with limited, local, person-to-person secondary transmission (*3*).

Although dromedary camels are considered to be the probable source for zoonotic infections in humans, the mode of transmission from animals to humans is not understood (*4*). In 2014, Saudi Arabia experienced an outbreak due to increased zoonotic transmission and amplification by health care–related human-to-human transmission (*3*); the risk for secondary transmission from patients to household contacts was estimated at ≈5% (*5*). To prevent secondary cases and local transmission, the World Health Organization recommends monitoring all contacts of confirmed patients (*6*). 

On May 13 and 14, 2014, MERS-CoV infection was confirmed in 2 residents of the Netherlands who had taken pilgrimages to Medina and Mecca, Saudi Arabia (*7*). We undertook comprehensive monitoring of contacts of these patients and evaluated the risk for secondary transmission and the effects of the monitoring on the contacts.

## The Study

Formal ethical approval from a medical ethical committee was not required for this research because it was carried out as part of the public health monitoring and evaluation of contacts and did not entail subjecting participants to medical treatment. From the onset of symptoms in the 2 MERS-CoV patients (May 1) until their discharge from the hospital (June 5), they came into contact with 131 persons. Of these, 78 had unprotected exposure (defined as >15 min of face-to-face contact without wearing personal protective equipment) and 53 had protected exposure (defined as providing care while wearing adequate personal protection at all times). Of the unprotected contacts, 29 were members of the patients’ travel group, 17 were aircraft contacts, and 32 were contacts in the Netherlands before hospital admission (28 relatives plus 4 persons at a general medical practice and the hospital emergency department, including 1 health care worker). The travel group had traveled with the 2 confirmed case-patients through Saudi Arabia during April 26–May 10 and had direct contact with them. Four travelers reported direct contact with dromedary camels, 11 consumed unpasteurized camel milk, and 4 visited a local hospital. One traveler accompanied 1 case-patient to 4 different hospitals and shared a hotel room with both case-patients (*7*). The aircraft contacts had been seated within 3 rows of the case-patients on the return flight.

All contacts were asked to take their temperature twice a day and report any episode of fever (temperature >38°C), cough, diarrhea, or dyspnea for 14 days following their last possible exposure to the case-patients. Unprotected contacts were asked to remain in the country during the monitoring period. Throat swabs were obtained from contacts on days 7 and 14 postexposure, and serum samples were drawn on days 7 and 21 postexposure (Technical Appendix). Throat swab samples from 1 relative contact were unavailable; a second serum sample was missing from 7 relatives, 3 aircraft contacts, and 1 travel contact (a woman who had had no contact with animals, had not visited a hospital, and had no concurrent conditions). Eight contacts who reported symptoms (7 unprotected and 1 protected) were sampled immediately after onset of symptoms. MERS-CoV reverse transcription PCR (RT-PCR) was performed on paired throat swabs from 106 (81%) and serologic analysis on paired serum samples from 99 (76%) of the 131 contacts (Table 1). PCR did not detect MERS-CoV RNA from any throat swab or serum samples, and MERS-CoV–specific IgG responses were absent in serum samples tested (*8*) (Table 1). All specimens obtained from the symptomatic contacts tested negative by RT-PCR and analysis of paired serum samples for MERS-CoV. 

**Table 1:** Laboratory results and compliance of follow-up among 131 unprotected and protected contacts of 2 patients with imported MERS-CoV infections, the Netherlands, 2014*

Type of contact	No. persons	Male sex, %	Median age, y (range)	No. (%) contacts				
				First throat swab sample	Paired throat swab sample	First serum sample	Paired serum sample	Symptomatic
Unprotected contacts	78	40	45 (1–78)	77 (99)	77 (99)	77 (99)	67 (86)	7 (9)
Travel group	29	45	52 (9–70)	29 (100)	29 (100)	29 (100)	28 (97)	2 (7)
Aircraft contacts	17	47	39 (7–78)	17 (100)	17 (100)	17 (100)	14 (82)	2 (12)
Other contacts†	32	32	44 (1–64)	31 (97)	31 (97)	31 (97)	25 (78)	3 (9)
Protected contacts	53	34	36 (18–63)	44 (83)	29 (55)	53 (100)	32 (60)	1 (2)
Total contacts	131	37	41 (1–78)	121 (92)	106 (81)	130 (99)	99 (76)	8 (6)

*MERS-CoV, Middle East respiratory syndrome coronavirus. †Other contacts were those who had contact with the case-patients after their return to the Netherlands: 28 relatives, plus 4 persons at a general medical practice and the hospital emergency department, including 1 health care worker.

All contacts also received an online questionnaire containing questions about demographics, type of contact, quality of information received, perceived severity and vulnerability, feelings of anxiety, interference of the measures with daily life, and knowledge of the measures and travel advice (Technical Appendix). To evaluate the effect of monitoring, we used the Revised Impact of Event Scale (IES-R), a validated questionnaire designed to assess current subjective distress for a specific traumatic life event (*9*). The IES-R contains 22 items divided into 3 subscales: avoidance (e.g., avoidance of feelings), intrusion (e.g., nightmares) and hyperarousal (e.g., anger). The mean score on 3 subscale domains indicates the level of distress experienced (*9*). Mean scores of unprotected contacts were compared with those of protected contacts by a Wilcoxon rank-sum test or *t*-test. Significance was determined at the 5% level (p value <0.05). A total subjective stress IES-R score with a maximum score of 88 (Likert scale of 0–4 [0, never; 1, seldom; 2, sometimes; 3, often; 4, very often]) can be calculated. We considered a score >20 to be an indicator of posttraumatic stress disorder to enable comparison with previous studies (*10**,**11*).

Of 131 contacts, 72 (55%, 48 unprotected and 24 protected) filled out the questionnaire. The median age was 39 years (range 9–77 years); 53% were female, and 51% had at least a college education. Protected contacts were younger (median of 31 vs. 48 years) and had a higher education (88% vs. 31%) than unprotected contacts. The mean IES-R score of all contacts was 7.9 (95% CI 5.5–10.3); the score was >20 for 16 (22%) contacts. Unprotected contacts had a significantly higher mean IES-R score (10.4 95% CI 7.2–13.6 versus 2.9, 95% CI 0.6–5.3); this result was also seen on the different subscale domains (Table 2).

**Table 2:** Results of survey assessing psychological effects of monitoring among 72 contacts of 2 patients with imported MERS-CoV infections, stratified by unprotected versus protected contacts, the Netherlands, 2014*

Category		Mean IES-R score (95% CI)	
	All contacts	Unprotected contacts	Protected contacts
Total IES-R score	7.9 (5.5–10.3)	10.4 (7.2–13.6)	2.9 (0.6–5.3)
Avoidance	2.2 (1.3–3.1)	3.1 (1.8–4.3)	0.5 (−0.04–1.1)
Intrusion	3.4 (2.5–4.4)	4.3 (3.1–5.5)	1.8 (0.5–3.0)
Hyperarousal	2.0 (1.3–2.7)	2.7 (1.7–3.6)	0.6 (−0.04–1.3)

*IES-R, Revised Impact of Event Scale.

## Conclusions

We monitored 131 contacts of 2 case-patients with imported MERS-CoV infections in the Netherlands. Laboratory testing did not indicate transmission of the virus, including among contacts with high-risk exposures or those who developed respiratory symptoms. We also found no infections among travelers from the same group. Our findings agree with reports from Greece and Italy, in which no and limited secondary transmission, respectively, was found among close contacts of MERS-CoV patients (*12**,**13*)*.*

Survey results show a substantial psychological effect of monitoring on contacts, especially unprotected contacts. As with other emerging infections, such as Marburg hemorrhagic fever and severe acute respiratory syndrome, quarantine or monitoring of contacts leads to psychological distress, measured by high IES-R scores (*10**,**11**,**14*). When stratifying by type of contact, the total mean IES-R score and the subscale scores were highest for unprotected contacts—those with the highest risk for exposure. We found increased symptoms of posttraumatic stress disorder in a considerable number of contacts, similar to findings by Hawryluck et al. (*11*) and Reynolds et al. (*10*).

The survey response rate of 55% limits interpretation of results; motives for noncompliance remain unknown. Also, recall bias might influence recollection of experiences. Besides exposure, monitoring has contributed to the psychological effect. Whether the number of questions induced stress is not known, but participants did not mention this as a concern.

Our findings illustrate the feasibility of comprehensive follow-up of contacts of MERS-CoV patients and clarify the risk for asymptomatic secondary transmission. The psychological effect of contact monitoring and the observed low rates of MERS-CoV transmission in several studies, including this investigation, indicate that thoughtful but limited assessment of close contacts is prudent. Identification of close contacts of those who are infected should be carefully considered, and decisions about monitoring and testing of contacts should be made primarily on the basis of clinical and epidemiologic risk factors.

**Technical Appendix.** Laboratory methods used regarding Middle East respiratory syndrome coronavirus and the results of the questionnaire study undertaken to assess contacts’ knowledge, quality of information, perceptions of severity and vulnerability, and interference of contact monitoring measures with daily life, the Netherlands.
