# Supplementary material for: Follow-up of Contacts of Middle East Respiratory Syndrome Coronavirus–Infected Returning Travelers, the Netherlands, 2014
Source: Emerg Infect Dis. 2015 Sep;21(9):1667–9. doi: 10.3201/eid2109.150560 (PMC4550153; doi:10.3201/eid2109.150560)
Supplement: Supplementary file 1 — Technical Appendix. Laboratory methods used regarding Middle East respiratory syndrome coronavirus and the results of the questionnaire study undertaken to assess contacts’ knowledge, quality of information, perceptions of severity and vulnerability, and interference of contact monitoring measures with daily life, the Netherlands. [file 15-0560-Techapp-s1.pdf]

Article DOI: http://dx.doi.org/10.3201/eid2109.150560

Follow-up of Contacts of Middle East Respiratory Syndrome Coronavirus–Infected Returning Travelers, the Netherlands, 2014

Technical Appendix

Part 1: Laboratory methods for PCR detection of MERS-CoV and antibody detection

Total RNA was extracted from 200 µL Universal Transport medium (COPAN) by using the High Pure RNA isolation kit (Roche, Mannheim, Germany) and tested for MERS-CoV RNA by internally controlled real-time reverse transcription PCR targeting Orf1A, nucleocapsid, and UpE with the TaqMan Fast Virus 1-Step Master Mix (Applied Biosystems, Bleiswijk, the Netherlands) as described (*1*–*3*). The results were independently confirmed in 2 laboratories (RIVM and Erasmus MC) and samples were considered MERS-CoV positive when at least 2 different MERS-CoV specific targets were reactive (*4*).

Serum samples were tested in at a 1:20 dilution for IgG reactive with MERS-CoV (residues 1–747), severe acute respiratory syndrome–CoV (residues 1–676) and human coronavirus OC43 (residues 1–760) spike domain S1 antigens by using extensively validated protein-microarray technology, as described (*3*,*5*). Confirmation was performed by using a neutralization assay (*4*).

Part 2: Questionnaire to assess knowledge, quality of information, perceptions of severity and vulnerability and interference of measures with daily life

All contacts received an invitation by post including a link and a unique code to access an online questionnaire (Formdesk, Innovero Software Solutions B.V., The Hague, The Netherlands). The questionnaire contained precoded questions on demographics, type of contact, quality of information received, perceived severity and vulnerability, feelings of anxiety, perceived interference with daily life, and knowledge (including questions regarding travel advice for the travel group). The questionnaire was based on questionnaires used in

similar studies on severe acute respiratory syndrome, infection with avian influenza, infection with influenza A (H1N1) virus, and Marburg hemorrhagic fever (*6–10*), with some alterations.

Questions on perceived severity and vulnerability, feelings of anxiety, and perceived interference with daily life (e.g., restrictions on social life and fear of becoming infected) were based on an integrated model designed to explain health behavior (*11,12*). Knowledge of MERS-CoV was examined with 7 true/false/don't know statements. The members of the travel group (n = 29) were also asked to answer questions regarding the travel advice they had received before their trip to Saudi Arabia. The presence of concurrent conditions and use of medicines were not part of this questionnaire, but were addressed in another study and published elsewhere (*13*). The questionnaire took ≈15 min to fill out and the information was processed anonymously.

Data Analysis

Differences in knowledge, impact of monitoring measures, quality of information, and perception between unprotected and protected contacts were compared in contingency tables by using the χ^2 test. For assessing knowledge, a summary score was created on the basis of the number of correct answers (range 0–7). Significance was determined at the 5% level (p-≤0.05). Data analysis was performed with SAS 9.3(SAS Institute, Cary, NC, USA).

Results

Demographics

Of the 131 contacts, 72 (55%) filled out the questionnaire. Among the unprotected contacts, the response rate was highest for the travel group (22 [76%] of 29), compared with 19 (59%) of 32 for the other unprotected contacts and 7 (41%) of 17 for the aircraft contacts. Among the protected contacts the response rate was 24 (45%) of 53. The median age of respondents was 39 years (range 9–77 years), 53% were female, and 51% had at least a college education. Protected contacts were younger (median of 31 years vs. 48 years) and had more education (88% bvs. 31%) than unprotected contacts.

Knowledge of MERS-CoV

Most (83%) contacts were aware of the symptoms related to MERS-CoV infection and knew that MERS-CoV is not common in the Netherlands (83%) (Table 1). In total, 76% of

the contacts knew that MERS-CoV could spread by having contact with a camel. Half of the contacts knew MERS-CoV can be transmitted from person to person. Half of the respondents knew that there is no specific treatment for MERS-CoV; one-third (36%) thought a vaccine was available. On the knowledge sum score, protected contacts (5.1, 95% CI 4.5–5.6) scored significantly higher than unprotected contacts (3.8, 95% CI 3.3–4.3).

Perception of MERS-CoV and Interference of the Measures with Daily Life

Perception

Most contacts (n = 54; 75%) perceived MERS-CoV as being (very) serious (Table 2). In addition, 69% of contacts (n = 50) thought MERS-CoV would have a (very) negative impact on their health. However, only 49% (n = 35) of the persons thought about MERS-CoV (very) often in the last month. Unprotected contacts thought significantly more often about MERS-CoV than did protected contacts (p = 0.02).

Interference of the Measures with Daily Life

Only 4% (n = 3) of contacts regularly perceived measuring or reporting their temperature as a burden. Most contacts (90%, n = 65) were not planning to leave the Netherlands and 93% experienced no problems with this measure. Extra costs were involved for 21 (29%) of the contacts. Being identified as a contact caused anxiety in respondents, and 38 (53%) of them were afraid of contracting MERS-CoV or infecting their families (53%, n = 38). These numbers were higher for unprotected than protected contacts (69%, [n = 33] vs. 21%, [n = 5] and 65%, [n = 31] vs. 29%, [n = 7]) respectively. Furthermore, approximately one-third of the contacts reported that their family members expressed anxiety about becoming infected (35%, n = 25). There were no protected contacts who felt seriously limited in their social contacts because of the measurements they had to take, compared with 16 unprotected contacts (22%) who did feel limited.

Information on MERS-CoV

Written instructions with detailed information on the monitoring measures and their rationale were received by 53 (74%) of 72 respondents. Of these 53 respondents, 41 (77%) found the information to be clear, 33 (73%) complete, 30 (56%) unequivocal. Only 4 (8%) thought the information was confusing, and 2 (4%) thought it was redundant. In total, 25 (47%) thought the information was clear, complete, and unequivocal.

Travel Advice

Twenty-three of the 29 participants to the pilgrimage trip to Saudi Arabia filled out this part of the questionnaire; 21 (91%) received travel advice or vaccinations before the trip (the other 2 were already vaccinated, for example against meningococcal disease and DTP, because of previous traveling). During the pretravel consultation, only 1 person received information on the possible transmission of MERS-CoV in the Middle East (avoid contact with animals, avoid drinking unpasteurized milk, and when having symptoms contact a doctor when returning to the Netherlands). However, although most did not receive any advice, 8 persons watched their health more carefully (35%), 9 reported that they were more compliant with personal hygiene measures during the trip (39%), 3 avoided contact with animals (13%), and 3 avoided contact with animals' waste (13%). Twelve did not change their behavior after receiving travel advice (52%).

References

1. Haagmans BL, Al Dhahiry SH, Reusken CB, Raj VS, Galiano M, Myers R, et al. Middle East Respiratory Syndrome coronavirus in dromedary camels: an outbreak investigation. Lancet Infect Dis. 2014;14:140–5. PubMed http://dx.doi.org/10.1016/S1473-3099(13)70690-X

2. Corman VM, Eckerle I, Bleicker T, Zaki A, Landt O, Eschbach-Bludau M, et al. Detection of a novel human coronavirus by real-time reverse-transcription polymerase chain reaction. Euro Surveill. 2012;17. PubMed

3. Corman VM, Muller MA, Costabel U, Timm J, Binger T, Meyer B, et al. Assays for laboratory confirmation of novel human coronavirus (hCoV-EMC) infections. Euro Surveill. 2012;17. PubMed

4. World Health Organization. Revised case definition for reporting to WHO–Middle East respiratory syndrome coronavirus [cited 2015 Mar 25]. http://www.who.int/csr/disease/coronavirus_infections/case_definition/en

5. Reusken CB, Haagmans BL, Muller MA, Gutierrez C, Godeke GJ, Meyer B, et al. Middle East Respiratory Syndrome coronavirus neutralising serum antibodies in dromedary camels: a comparative serological study. Lancet Infect Dis. 2013;13:859–66. PubMed http://dx.doi.org/10.1016/S1473-3099(13)70164-6

6. Reynolds DL, Garay JR, Deamond SL, Moran MK, Gold W, Styra R. Understanding, compliance and psychological impact of the SARS quarantine experience. Epidemiol Infect. 2008;136:997–1007. PubMed http://dx.doi.org/10.1017/S0950268807009156

7. Bults M, Beaujean DJ, de Zwart O, Kok G, van Empelen P, van Steenbergen JE, et al. Perceived risk, anxiety, and behavioural responses of the general public during the early phase of the Influenza A (H1N1) pandemic in the Netherlands: results of three consecutive online surveys. BMC Public Health. 2011;11:2. PubMed http://dx.doi.org/10.1186/1471-2458-11-2

8. van der Weerd W, Timmermans DR, Beaujean DJ, Oudhoff J, van Steenbergen JE. Monitoring the level of government trust, risk perception and intention of the general public to adopt protective measures during the influenza A (H1N1) pandemic in The Netherlands. BMC Public Health. 2011;11:575. PubMed http://dx.doi.org/10.1186/1471-2458-11-575

9. Vinck L, Isken L, Hooiveld M, Trompenaars M, Ijzermans J, Timen A. Impact of the 2009 influenza A(H1N1) pandemic on public health workers in the Netherlands. Euro Surveill. 2011;16. PubMed

10. Timen A, Isken LD, Willemse P, van den Berkmortel F, Koopmans MP, van Oudheusden DE, et al. Retrospective evaluation of control measures for contacts of patient with Marburg hemorrhagic fever. Emerg Infect Dis. 2012;18:1107–14. PubMed http://dx.doi.org/10.3201/eid1807.101638

11. Norman P, Boer H, Seydel E. Protection motivation theory. In: Conner M, Norman P, editors. Predicting health behaviour. Berkshire (UK): Open University Press; 2005. p. 81–126.

12. Champion V, Skinner C. The health belief model. In: Glanz K, Rimer BK, Viswanath K, editors. Health behavior and health education: theory, research, and practice. San Francisco: (CA): Jossey Bass; 2008. p. 45–66.

13. Fanoy EB, van der Sande MA, Kraaij-Dirkzwager M, Dirksen K, Jonges M, van der Hoek W, et al. Travel-related MERS-CoV cases: an assessment of exposures and risk factors in a group of Dutch travellers returning from the Kingdom of Saudi Arabia, May 2014. Emerg Themes Epidemiol. 2014;11:16. PubMed http://dx.doi.org/10.1186/1742-7622-11-16

Table 1. Middle East respiratory syndrome coronavirus general knowledge among contacts (n = 72) stratified by protected vs. unprotected contacts, the Netherlands 2014

Statement (correct answer)	All contacts (n = 72)		Unprotected contacts (n = 46)		Protected contacts (n = 26)		p value
	No. correct	% Correct	No. correct	% Correct	No. correct	% Correct	
Patients with MERS have a fever, are coughing, are short of breath, have difficulties breathing and have diarrhea (true)	60	83	37	77	23	96	0.04
MERS is prevalent in the Netherlands (false)	60	83	38	79	22	92	0.20
MERS is a bacterium causing severe lung disease (false)	13	18	6	13	7	31	0.08
In the Middle East MERS can be contracted through contact with camels or their products such as meat, milk, urine or feces (true)	55	76	34	71	21	88	0.11
MERS can be spread from person to person (true)	36	50	20	42	16	69	0.05
There is no specific treatment once you contract MERS (true)	36	50	24	50	12	50	1.00
There is a vaccine available for MERS (false)	46	64	48	56	19	81	0.06
Average number of correct answers (95% CI)	4.3 (3.8–4.7)		3.8 (3.3–4.3)		5.1 (4.5–5.6)		0.01

Table 2. MERS-CoV general perception among all contacts (n = 72) and stratified by unprotected- vs. protected contacts, the Netherlands 2014

Perception	Total (n = 72)		Unprotected contacts (n = 46)		Protected contacts (n = 26)		p value
	No.	%	No.	%	No.	%	
MERS is (very) serious to contract	54	75	34	71	20	83	0.3
MERS is (very) bad for my health	50	69	32	67	18	75	0.5
I have thought about MERS (very) often in the last month	35	49	28	60	7	29	0.02
